# Supplementary material for: Mutation analysis links angioimmunoblastic T-cell lymphoma to clonal hematopoiesis and smoking
Source: eLife. 2021 Sep 29;10:e66395. doi: 10.7554/eLife.66395 (PMC8480981; doi:10.7554/eLife.66395)
Supplement: Transparent reporting form [file elife-66395-transrepform1.docx]

***eLife’s* transparent reporting form**

We encourage authors to provide detailed information *within their submission* to facilitate the interpretation and replication of experiments. Authors can upload supporting documentation to indicate the use of appropriate reporting guidelines for health-related research (see [EQUATOR Network](http://www.equator-network.org/%20)), life science research (see the [BioSharing Information Resource](https://biosharing.org/)), or the [ARRIVE guidelines](http://www.plosbiology.org/article/info:doi/10.1371/journal.pbio.1000412) for reporting work involving animal research. Where applicable, authors should refer to any relevant reporting standards documents in this form.

If you have any questions, please consult our Journal Policies and/or contact us: [editorial@elifesciences.org](mailto:editorial@elifesciences.org).

**Sample-size estimation**

- You should state whether an appropriate sample size was computed when the study was being designed
- You should state the statistical method of sample size computation and any required assumptions
- If no explicit power analysis was used, you should describe how you decided what sample (replicate) size (number) to use

Please outline where this information can be found within the submission (e.g., sections or figure legends), or explain why this information doesn’t apply to your submission:

Sample size (numbers of patients and primary lymphoma/BM or PB pairs) was not determined with any statistical power analysis when this study was initiated, as the main goal of this study is to perform mutation analyses of these samples only. In addition, we simply collected as many as possible paired primary tumor and BM/PB samples in patients with AITL or PTCL-NOS (relatively rare lymphomas), so we decided the sample size based on the availability of the evaluable paired samples. As far as we know, the current study cohort (n=27, including 25 AITL cases and 2 PTCL-NOS cases, which was stated in Page #6 and #11 in the manuscript) is the largest reported to date.

**Replicates**

- You should report how often each experiment was performed
- You should include a definition of biological versus technical replication
- The data obtained should be provided and sufficient information should be provided to indicate the number of independent biological and/or technical replicates
- If you encountered any outliers, you should describe how these were handled
- Criteria for exclusion/inclusion of data should be clearly stated
- High-throughput sequence data should be uploaded before submission, with a private link for reviewers provided (these are available from both GEO and ArrayExpress)

Please outline where this information can be found within the submission (e.g., sections or figure legends), or explain why this information doesn’t apply to your submission:

To compare the mutation landscape, each sequenced primary lymphoma or matched BM/PB sample technically represents a biological replicate for the corresponding group. Processed sequencing data can be found in Supplementary file 3. The original DNA sequencing data (fastq files) will be uploaded into NCBI SRA database. The upload process has not been completed due to NCBI server issues, and we will complete this process after the server returns to normal).

**Statistical reporting**

- Statistical analysis methods should be described and justified
- Raw data should be presented in figures whenever informative to do so (typically when N per group is less than 10)
- For each experiment, you should identify the statistical tests used, exact values of N, definitions of center, methods of multiple test correction, and dispersion and precision measures (e.g., mean, median, SD, SEM, confidence intervals; and, for the major substantive results, a measure of effect size (e.g., Pearson's r, Cohen's d)
- Report exact p-values wherever possible alongside the summary statistics and 95% confidence intervals. These should be reported for all key questions and not only when the p-value is less than 0.05.

Please outline where this information can be found within the submission (e.g., sections or figure legends), or explain why this information doesn’t apply to your submission:

The statistical analysis conducted is described in the references to figures along the text in the Results section and the Materials and Methods section, as well as each figure legend.

(For large datasets, or papers with a very large number of statistical tests, you may upload a single table file with tests, Ns, etc., with reference to sections in the manuscript.)

**Group allocation**

- Indicate how samples were allocated into experimental groups (in the case of clinical studies, please specify allocation to treatment method); if randomization was used, please also state if restricted randomization was applied
- Indicate if masking was used during group allocation, data collection and/or data analysis

Please outline where this information can be found within the submission (e.g., sections or figure legends), or explain why this information doesn’t apply to your submission:

For the mutation analysis and comparison, the samples were categorized into primary lymphoma group (lymph node) and matched marrow or peripheral blood group. For the biomarker study, the patients were assigned into two different groups based on the TET2 mutation status as described in details in the Results section (Page #20).

**Additional data files (“source data”)**

- We encourage you to upload relevant additional data files, such as numerical data that are represented as a graph in a figure, or as a summary table
- Where provided, these should be in the most useful format, and they can be uploaded as “Source data” files linked to a main figure or table
- Include model definition files including the full list of parameters used
- Include code used for data analysis (e.g., R, MatLab)
- Avoid stating that data files are “available upon request”

Please indicate the figures or tables for which source data files have been provided:

Supplementary file 1

Supplementary file 2

Supplementary file 3

Supplementary file 4
